# Sulforaphane-Loaded Hydrogel Prolongs Fully MHC-Mismatched Skin Allograft Survival

**DOI:** 10.1021/acsabm.5c01022

**Published:** 2025-10-29

**Authors:** Lorena Doretto-Silva, Laura Quadros-Pereira, Anderson F. Sepulveda, Victor Y. Yariwake, José A. O. Nery-Neto, Eloisa M. da Silva, Isabela L. Doretto, Niels O. S. Câmara, Daniele R. de Araujo, Vinicius Andrade-Oliveira

**Affiliations:** † Mucosal Health and Immunity Laboratory (MHIL), Center for Natural and Human Science, 28105 Federal University of ABC, Santo André, São Paulo 09280-560, Brazil; ‡ SISLIBIO, Center for Natural and Human Science, Federal University of ABC, Santo André, São Paulo 09280-560, Brazil; § Institute of Biomedical Sciences, University of São Paulo, São Paulo 05508-000, Brazil; ∥ Paulista School of Medicine, Federal University of São Paulo, São Paulo 04021-001, Brazil; ⊥ Agronomy and Veterinary Medicine School, University of Brasília, Brasília 70910-900, Brazil; # Department of Biophysics, Paulista Medical School, Federal University of São Paulo, São Paulo 04021-001, Brazil

**Keywords:** acute rejection, transplant, sulforaphane, hydrogel, drug delivery

## Abstract

Despite immunosuppression, acute rejection (AR) is still a common setback among transplantation patients and is a risk factor for graft survival. Sulforaphane (SFN), a phytochemical present in crucifers, has been shown to have anti-inflammatory and immunoregulatory properties, yet its influences on immune cell activation as well as in graft survival are still unknown. Thus, the aim was to evaluate SFN’s effect, and to improve efficacy, efficiency, and availability, it was incorporated into thermosensitive polymeric hydrogels, to prevent AR in skin transplant (Tx) model mice. A thermosensitive hydrogel containing SFN (0.1%) and hyaluronic acid (HA) (0.5%) dispersed in a poloxamer matrix (PL407 at 20% w/v) was developed (GS-PL407 20%, HA and SFN) and characterized as a liquid-viscous hydrogel. The fully MHC-incompatible skin Tx procedure was performed by using donor skin Balb/c mice, transplanted into C57BL/6 recipient mice. The cytotoxicity test of GS was performed using in vitro assays with bone marrow-derived dendritic cells (BMDC). Treatment of BMDC with GS for 24 h presents no cytotoxicity. Untreated allograft mice present 100% of graft loss at day 9 post Tx. Remarkably, subcutaneous GS injection every 3 days promoted 80% of allograft survival for more than 14 days when compared with untreated recipients (*p* < 0.001). Histological analysis showed a lower level of inflammatory cells in the skin Tx of GS-treated mice. Flow cytometry analysis of draining lymph nodes at day 5 post Tx revealed that GS treatment reduced the frequency of DC and subtypes and function of the CD4^+^ T cells. In vitro GS-treated lipopolysaccharide-activated BMDC present less activation of costimulatory markers. Taken together, GS treatment reduced immune cell activation, postponing AR onset and prolonging allograft survival in Tx animals. This strategy highlights the role of SFN as a promising candidate for further studies in organ transplantation experiments and being tested combined with current immunosuppression protocols.

## Graphical abstract

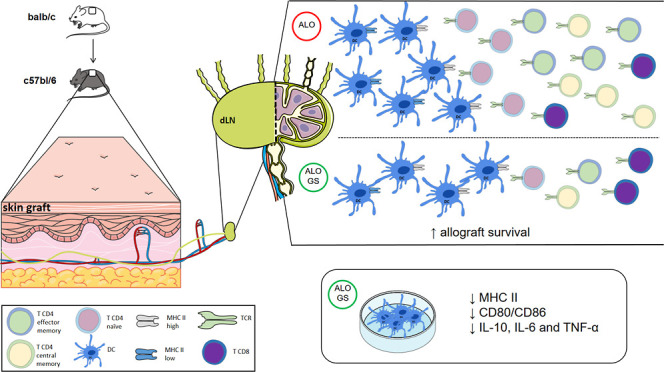

## 1 Introduction

One of the main challenges in transplantation (Tx) is managing acute rejection (AR),^1^ which still affects 5–20% ^2–5^ of patients despite current immunosuppressive strategies and emerging technologies such as gene editing utilizing CRISPR/Cas9 technology to xenotransplantation.^6^ AR is a complex immune response, primarily mediated by T lymphocytes and antigen-presenting cells (APCs), particularly dendritic cells (DCs),^7^ which initiate inflammation and graft damage through direct and indirect antigen presentation pathways.^8,9^

Although immunosuppressive drugs like corticosteroids, rapamycin, and calcineurin inhibitors are commonly used,^10–14^ they are not always effective in restoring full organ function or preventing long-term rejection. This has led to a growing interest in immunomodulatory approaches that target specific immune cells, especially DCs and T lymphocytes, to induce tolerance and improve graft survival.^15^

Sulforaphane (SFN), a natural isothiocyanate derived from cruciferous vegetables,^16–18^ has shown anti-inflammatory, antioxidant, and immunomodulatory properties in various disease models.^19–23^ It can influence DC and T-cell function^24^ and has demonstrated therapeutic potential in nonsolid organ transplantation models.^25^ However, its clinical application is limited by its short half-life and instability.^26^

To approach these limitations, thermosensitive hydrogels based on biocompatible polymers such as poloxamers,^27–29^ mainly those made up of PL 407 (PL407-Pluronic F-127).^27,30–35^ To enhance these systems, modifications can be made by combining PL with compounds like hyaluronic acid (HA), a key extracellular matrix component with lubricating and viscosupplementation properties, when presented with high molar masses.^36–38^ The combination of PL407 and HA creates a biocompatible, biodegradable drug delivery system,^39,40^ capable of encapsulating SFN, improving its stability and controlled release, and suggesting therapeutic efficacy.^41^

The structure of the formulation is already known, but its immunomodulatory behavior in the Tx application is still unknown. This article will enable the application of therapeutic approaches for the treatment of AR in Tx, both in the description of the molecular effects and in the development of new pharmaceutical formulations based on thermosensitive hydrogels, which allow a controlled release of the phytochemical, in addition to advancing the knowledge of the signaling pathways activated with the SFN, in models in which acute and prolonged inflammation has deleterious effects.

## 2 Results

### 2.1 Hydrogel Physicochemical Characterization

The hydrogels were prepared by direct dispersion of SFN (1 mg/mL) in PL407 solutions at concentrations of 20% or 25% w/v in ultrapure water, forming hybrid systems with HA at concentrations of 0.5% w/v. DSC and rheology data of isolated compounds, PL407 solutions, with and without HA, in the presence and absence of SFN, are presented in Table S1. Temperature sweeps (Figure 1A) show in both GS formulations a sol–gel transition temperature higher than room temperature (∼23 °C) and lower than body temperature (∼36,5 °C), indicating the formation of structured hydrogels close to physiological temperature, allowing an efficient drug release. In frequency sweeps (Figure 1A), the predominance of the elastic module G′ over the viscous module *G*″ is observed, which ensures the maintenance of the gel structure even in mechanical oscillation. Furthermore, the analysis of the flow curves (Figure 1B) demonstrates that the materials can return to their initial conformation, indicating stability in the passage of the material through the needle bevel and stability in the application.^30^

**Figure 1:**
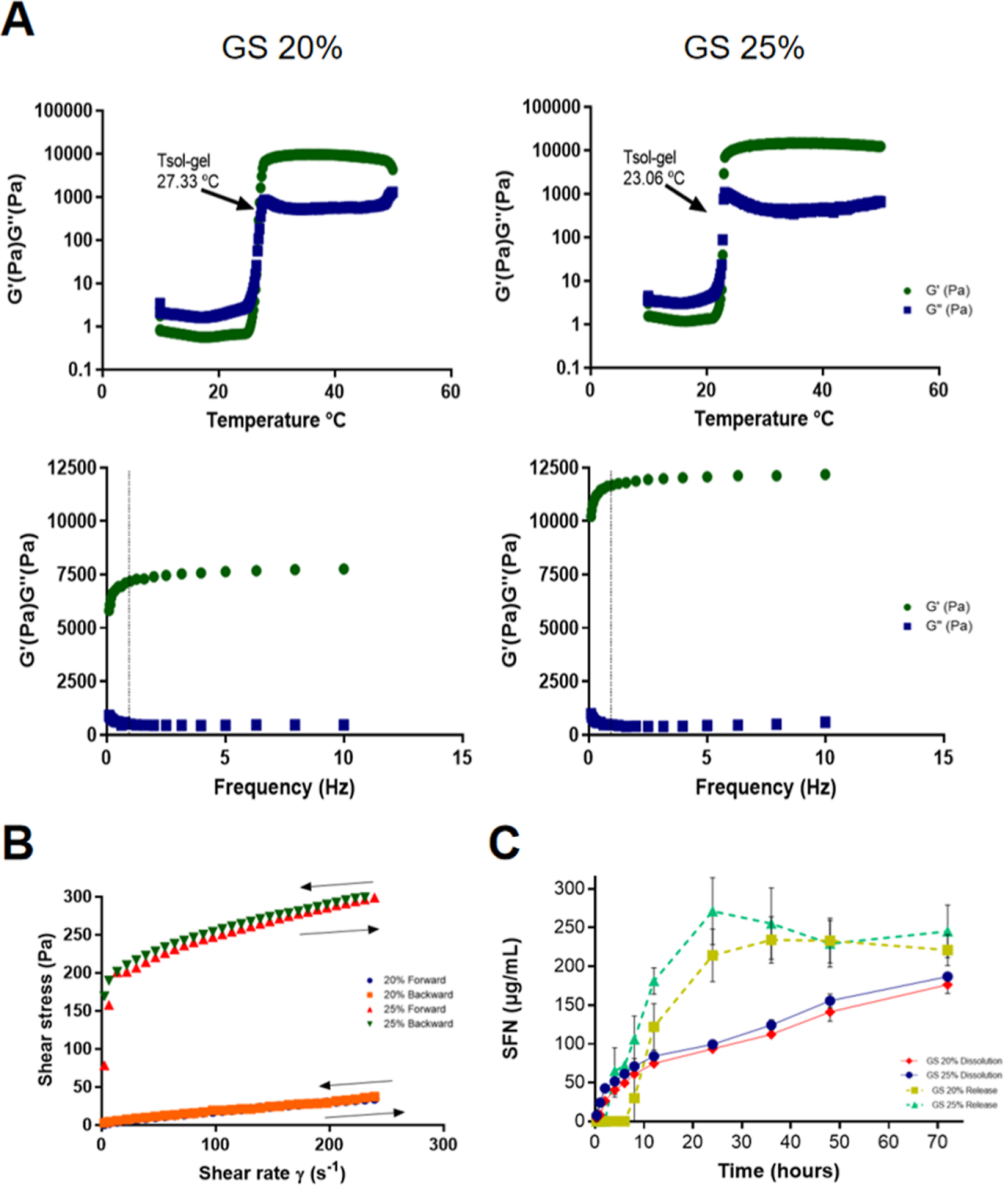
Rheology and release profiles’ characterization for PL407 (20%) and PL407 (25%) with HA (0.5%) and SFN (0.1%) analysis. (A) Representative rheograms for GS 20% and GS 25%. (B) Rheological flow curves for GS 20% and GS 25%. (C) Profiles of SFN released and dissolution for GS 20% and GS 25% after 30 min to 72 h. Arrows represent sol–gel transition temperatures (Tsol–gel). *G*′, elastic modulus; *G*″, viscous modulus; GS, formulation containing hydrogel PL407, HA, and SFN. Data were expressed as mean ± SD from triplicate.

In vitro release and dissolution tests were performed to verify the profiles related to the dissolution and release processes of SFN from the hydrogel under a limiting membrane and direct contact with the environment. Thus, it was possible to verify the mechanisms acting in the drug release process, regarding its diffusion during release or erosion of the gel and dissolution.

The in vitro release kinetics of SFN (in 5 mM phosphate buffer, pH 7.4), by the release assay, were linear up to 24 h. The GS 20% formulation maintained the SFN release profile with concentration values of 214.3 ± 4.94 μg/mL (24 h) and 234 ± 4.31 μg/mL (36 h). On the other hand, the increase in the PL concentration, GS 25%, maintained the SFN release profile with concentration values between 271.8 ± 6.26 μg/mL (24 h) and 255 ± 6.66 μg/mL (36 h). In order to confirm that it continues to be released over time, the assays were performed for up to 72 h (Figure 1C). The dissolution tests showed linearity and a gradual increase in the concentration of SFN in the receiving medium for up to 72 h. Both formulations showed equivalence in the concentration values of released SFN (Figure 1C). Our data considered the use of the Korsmeyer–Peppas model, since there is a combination of two processes: diffusion and erosion, confirmed with the release exponent, n, with values > 1 (Supporting Information #1 and Table S2).^42^ The dissolution test showed linearity and a gradual increase in the SFN concentration in the receptor medium for up to 72 h. Putting together all characterization, GS 20% displays better dynamic behaviors for the application in a Tx model. Thus, from now on, all utilization of GS in vivo and in vitro was GS 20%.

### 2.2 GS Treatment in the Skin Tx Model

Before in vivo treatment, the cytotoxicity of the formulation was performed using flow cytometry, taking into account the different concentrations of SFN alone and associated with GEL, considering the labeling for live and dead (Figure S1). We observed that the concentrations of pure SFN presented a greater quantity of dead cells, but GS showed good cell viability (Figure S1).

The skin Tx model is a well-established model in the literature,^43,44^ and it was chosen as a model for solid organ transplantation due to the possibility of monitoring AR of the graft in a macroscopic and less invasive manner for the animal. To test the therapeutic effect of GS to prevent AR, animals underwent allogeneic Tx and were treated with subcutaneous injection (s.c.) containing GS, every 3 days for 14 days (Figure 2A). We observed that the animals that underwent Tx and were treated with GS showed a delay in the appearance of signs related to AR (Figure 2B). Analyzing the survival curve (Figure 2C), we observed that the animals in the control group without treatment, ALO, began to lose the graft on day 6 post Tx, and by day 9 post Tx, all lost their grafts. Notably, following the animals until day 14 post Tx, animals treated with GS present 80% graft survival.

**Figure 2:**
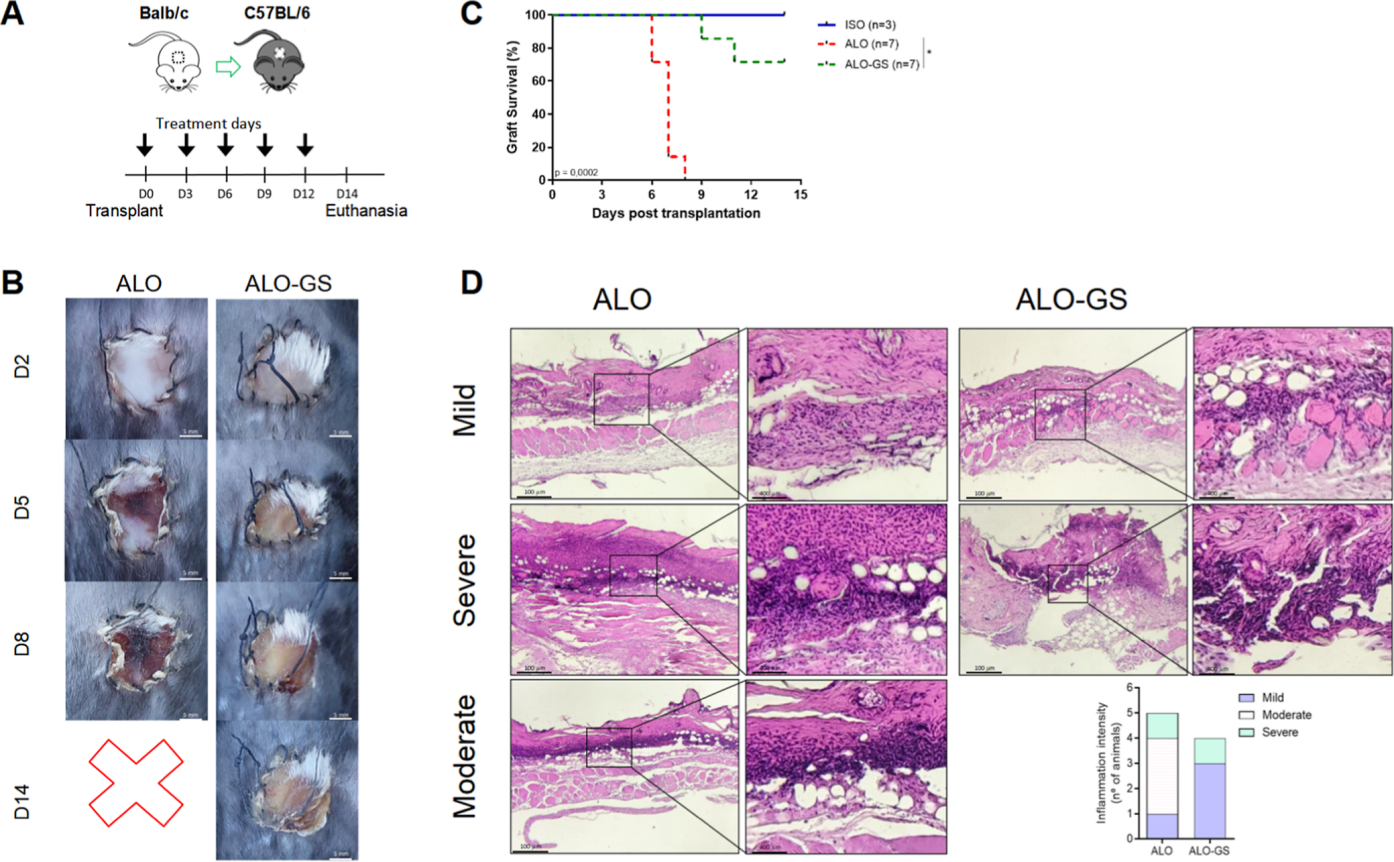
In vivo treatment with GS increases graft survival after skin allograft transplantation. (A) Illustration of the skin transplantation model from Balb/c donor mice into fully allogeneic C57BL/6 recipient mice, treated every 3 days for 14 days. (B) Graft survival photography monitoring 14th post Tx comparing the ALO group versus the ALO-GS-treated group. (C) Survival curve of graft progression for 14 days. (D) Histological HE slides of mouse skin grafts of ALO (*n* = 5) and ALO-GS (*n* = 4)-treated groups after 5 days post Tx, with characterization of the intensity of the inflammatory infiltrate, observed to be ranging from mild to moderate and severe. (Right) Histological view under 100X and (left) histological view under 400X. Intensity of inflammation considering the number of animals per group. HE, hematoxylin and eosin; ISO, isogenic group. ***p ≤ 0.001 for the Mantel–Cox test, comparing ALO versus ALO-GS group.

Given that ALO animals start to lose their grafts on day 6 (Figure 2C), we performed a new set of experimental groups and euthanized the animals on day 5 post Tx for cell and tissue collection and analysis to understand how GS treatment promotes prolonged graft survival. Histological slides present a less inflammatory infiltration in the GS-treated group compared to the untreated ALO (Figure 2D).

Flow cytometry analysis of draining lymph node (dLN) cells on the 5th day post Tx demonstrated that GS-treated animals promoted a light increase in DC CD86^+^ (CD11c^+^CD11b^+^) cell frequency and a decrease in CD80^+^ and CD86^+^ (CD11c^+^CD11b^+^) median fluorescence intensity (MFI) (Figure 3A), as well as a reduction in the MHC II^low^ population, compared to MHC II^high^ population (Figure 3B), suggesting that the treatment decreased the activity of the DC, and this may compromise its function as an APC once MHC and costimulatory markers are down.

**Figure 3:**
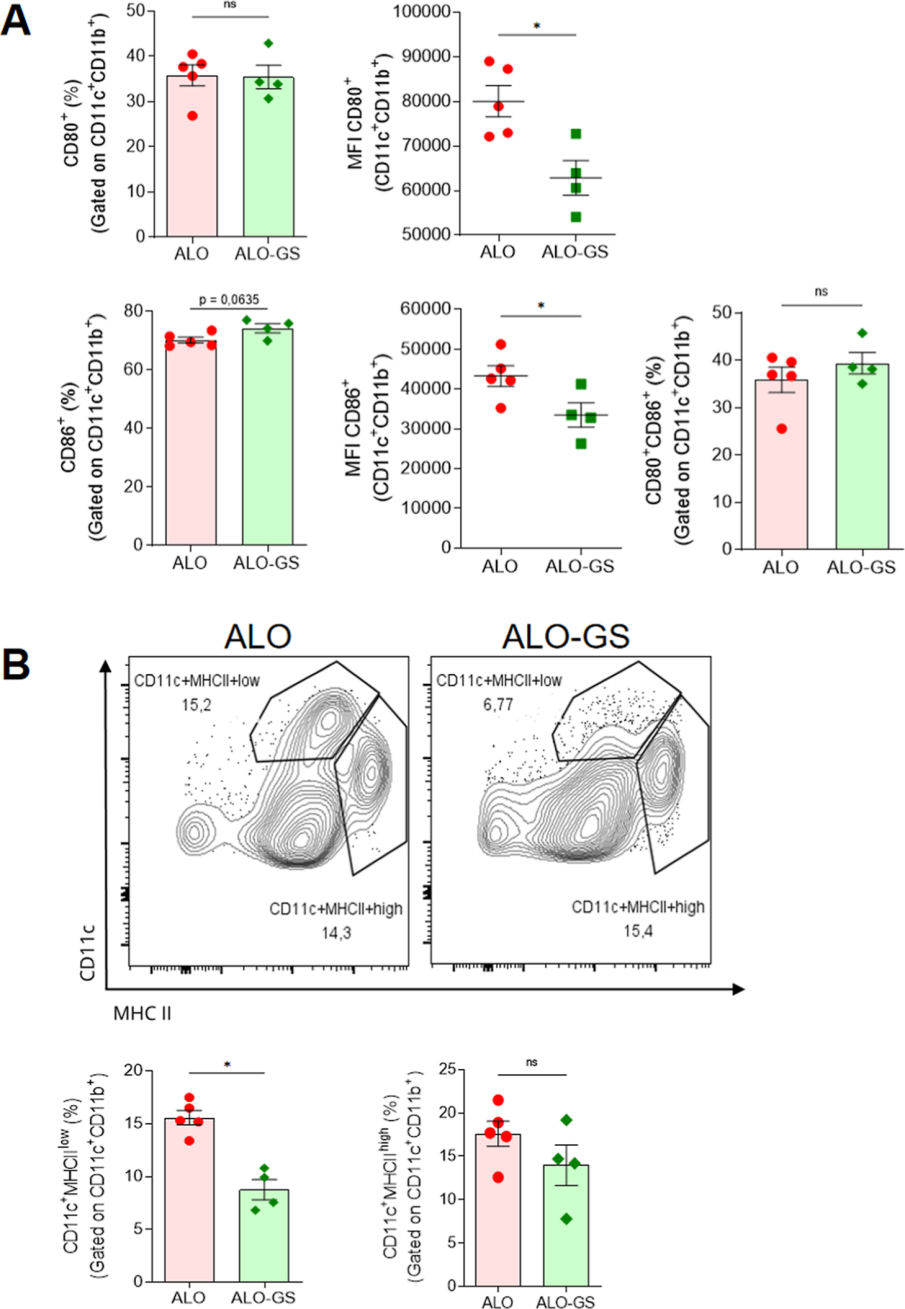
GS treatment reduces the frequency of DC population post Tx. C57Bl/6 animals received Balb/c skin and were treated with GS every 3 days. Animals were euthanized on day 5 post Tx to analyze the draining lymph node by flow cytometry. (A) Analysis of the percentage and MFI of costimulatory molecules, such as CD80^+^, CD86^+^, or CD80/86^+^ cells, considering the CD11c^+^CD11b^+^ gating strategy. (B) Representative cytometry panel of the frequency (above) of the control ALO and ALO-GS-treated groups. Quantification of the frequency of MHC II^+^ cells (below), considering the CD11c^+^CD11b^+^MHCII^high^ or MHC^low^ gating strategy. The Shapiro–Wilk test was performed for normality tests and the unpaired *t*-test for statistical differences. NS, non-significant; dLN, draining lymph node; MHC, major histocompatibility complex. *p-value ≤0.05.

Having seen overall inhibition of DC activation, next, we evaluated the frequency of CD4 and CD8 T lymphocyte subpopulations in the skin dLN of the graft 5 days post Tx. We observed that treatment with GS decreased the frequency of CD4^+^ T lymphocytes, but not CD8^+^ T lymphocytes (Figure 4A). Evaluating the different stages of differentiation of CD4^+^ T lymphocytes, we observed that treatment with GS reduced the frequency of all subpopulations of CD4^+^ T cells analyzed (naïve, effector memory, and central memory (Figure 4B,C), while we did not observe differences in these populations considering CD8^+^ T cells (Figure S2A,B).

**Figure 4:**
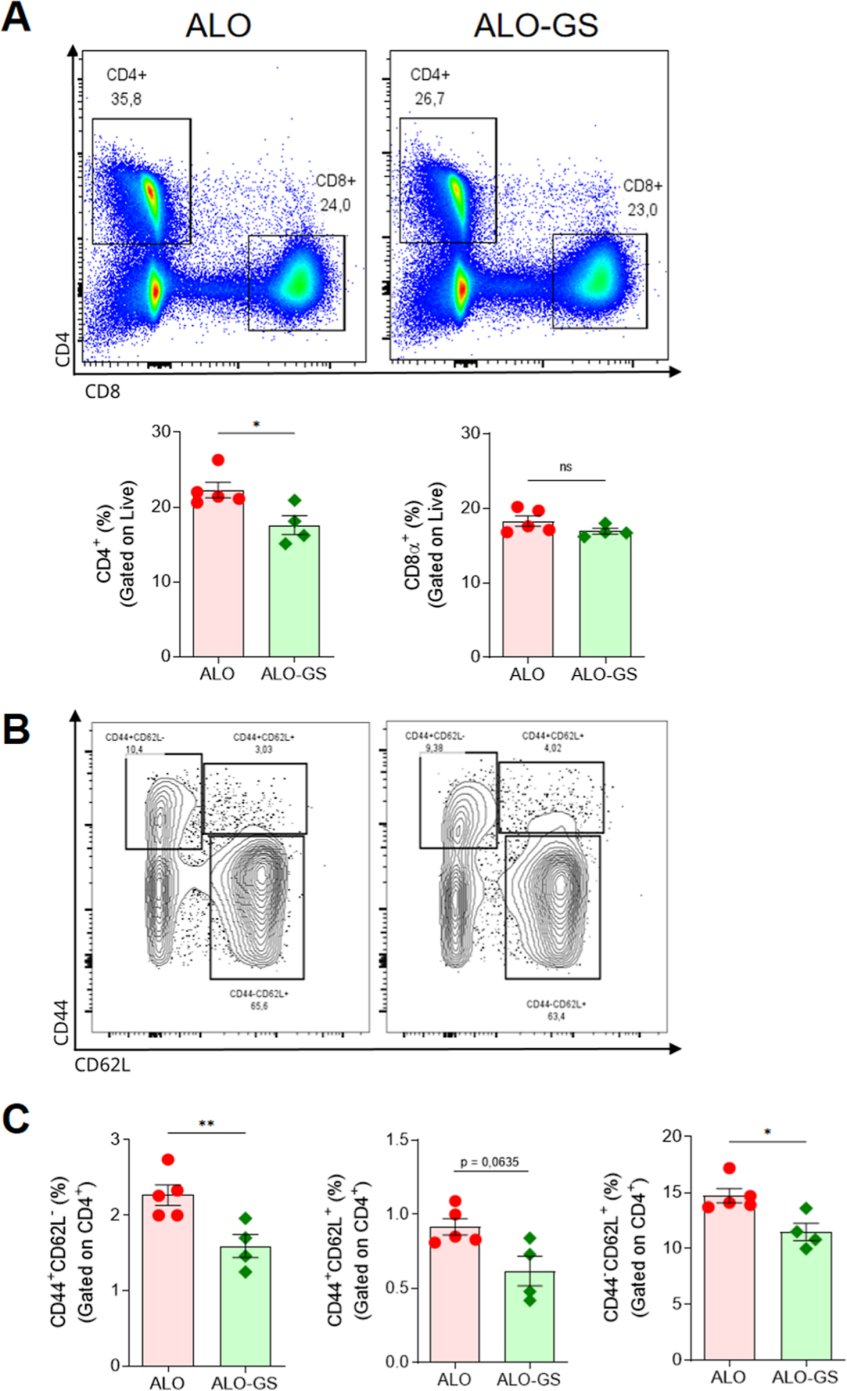
GS treatment reduces the frequency of T CD4 lymphocyte population in the dLN 5 days post Tx. (A) Representative cytometry panel for the frequency of parent and quantification of the frequency of CD4^+^ T and CD8^+^ T lymphocytes on live cells. (B) Representative cytometry panel for the frequency of parent and (C) frequency of effector memory (CD44^+^CD62L^–^), central memory (CD44^+^CD62L^+^), and naïve T cells (CD44^–^CD62L^+^) from CD4^+^ T lymphocytes. The Shapiro–Wilk test was performed for normality tests and the unpaired *t*-test for statistical differences. NS, non-significant; dLN, draining lymph node. *p-value ≤0.05; **p ≤ 0.01.

The next step was to evaluate the function of T cells in the dLN by looking for their ability to make cytokines. We observed that treatment with GS reduced the frequency of CD4^+^ T cells producing IFN-γ (Figure 5), but not IL-17. Furthermore, treatment with GS did not interfere with the production of IFN-γ and IL-17 by CD8^+^ T cells (Figure S3).

**Figure 5:**
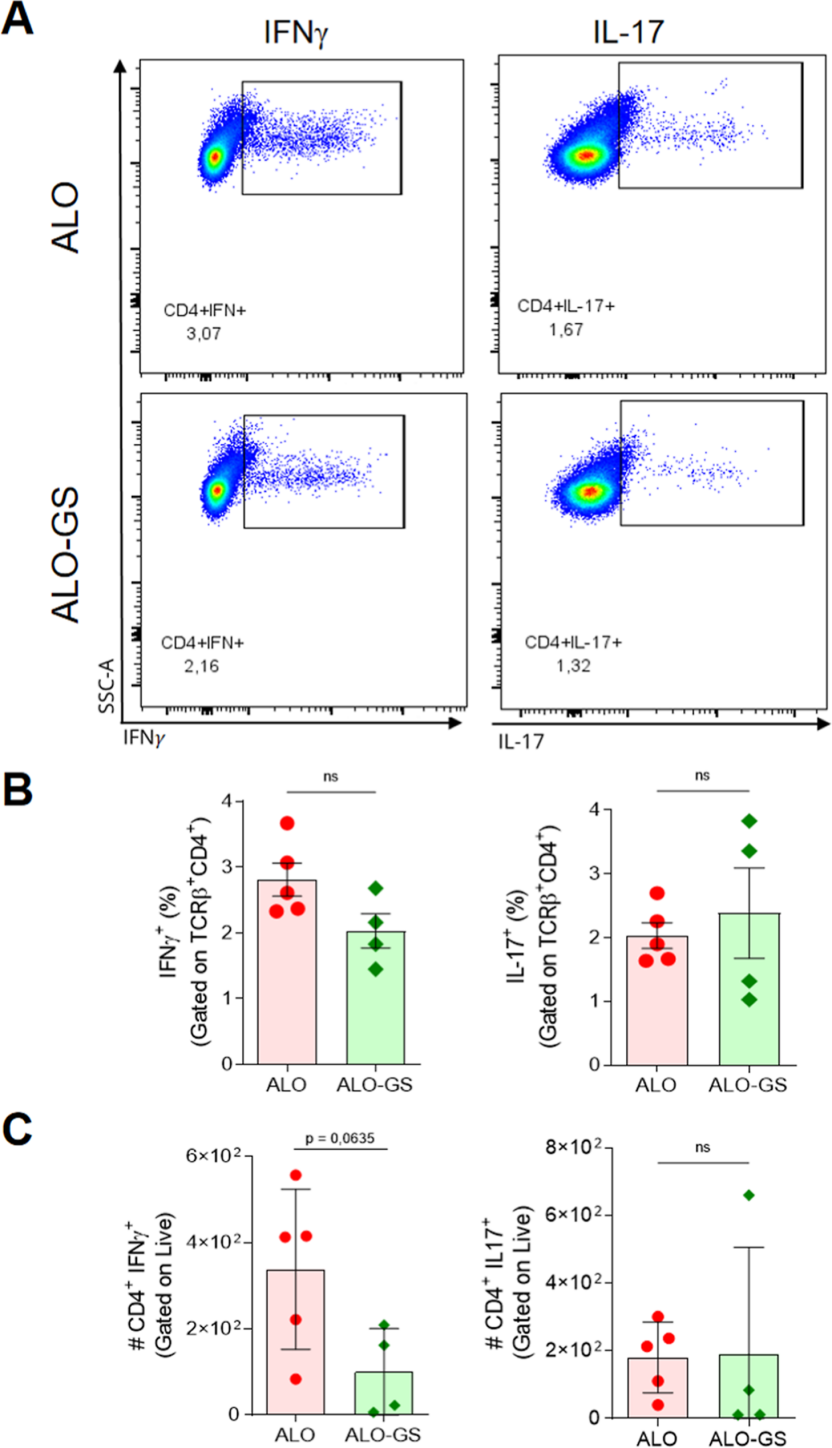
GS treatment reduced the frequency of IFN-γ-producing CD4^+^ T cells, but not IL-17, in the dLN 5 days post Tx. (A) Representative cytometry panel of the frequency of IFNγ^+^ and IL-17^+^ CD4^+^ T (gated on TCRγ^+^CD4^+^ cells). (B) Quantification of the frequency of production of cytokines IFNγ and IL-17 according to the values in (A). (C) The absolute number of CD4^+^, CD4^+^IFNγ^+^, or CD4^+^IL-17^+^ T cells relative to (B). The Shapiro–Wilk test was performed for normality tests and the unpaired *t*-test for statistical differences. NS, non-significant; IFNγ, interferon gamma; IL-17, interleukin 17; SSC-A, side scatter; dLN, draining lymph node.

### 2.3 GS Treatment in DC Culture

Taken together, these data suggest that GS treatment interferes with the activation of DCs in vivo and the differentiation and activation of CD4^+^ T lymphocytes, which may explain the prolongation of graft survival.

To ensure that GS treatment did not produce toxic effects on important organs, the serum was analyzed to supplement the formulation’s biosafety. Urea and creatinine assays showed no difference between their concentrations in the ALO and ALO-GS groups (Figure S4A). ALO-GS ALT/AST increased compared to the ALO group, but this was expected, considering their natural increase post transplant and initial hydrogel use (Figure S4B).

As the GS treatment modulated DC activation in vivo, we want to know whether GS treatment could also impact DC activation in vitro. For that, we generated bone marrow-derived dendritic cells (BMDC) for 6 days and treated them with SFN, GEL, or GS for 24 h, followed by stimulation with LPS (20 ng/mL) for another 24 h. The isolated SFN showed a low frequency of live cells (Figure 6A), but the isolated GEL and GS showed a good frequency compared to the LPS group. GS had a decrease in total MHC II^+^ and MHC II^high^, with no difference in MHC II^low^. Interestingly, MHC II^low^ was more frequent in the isolated treatment with GEL (Figure 6B). On the other hand, the costimulatory molecules CD80/CD86, in the GS and GEL groups, showed a decrease (Figure 6C) compared to the activated LPS group (see Figure 7).

**Figure 6:**
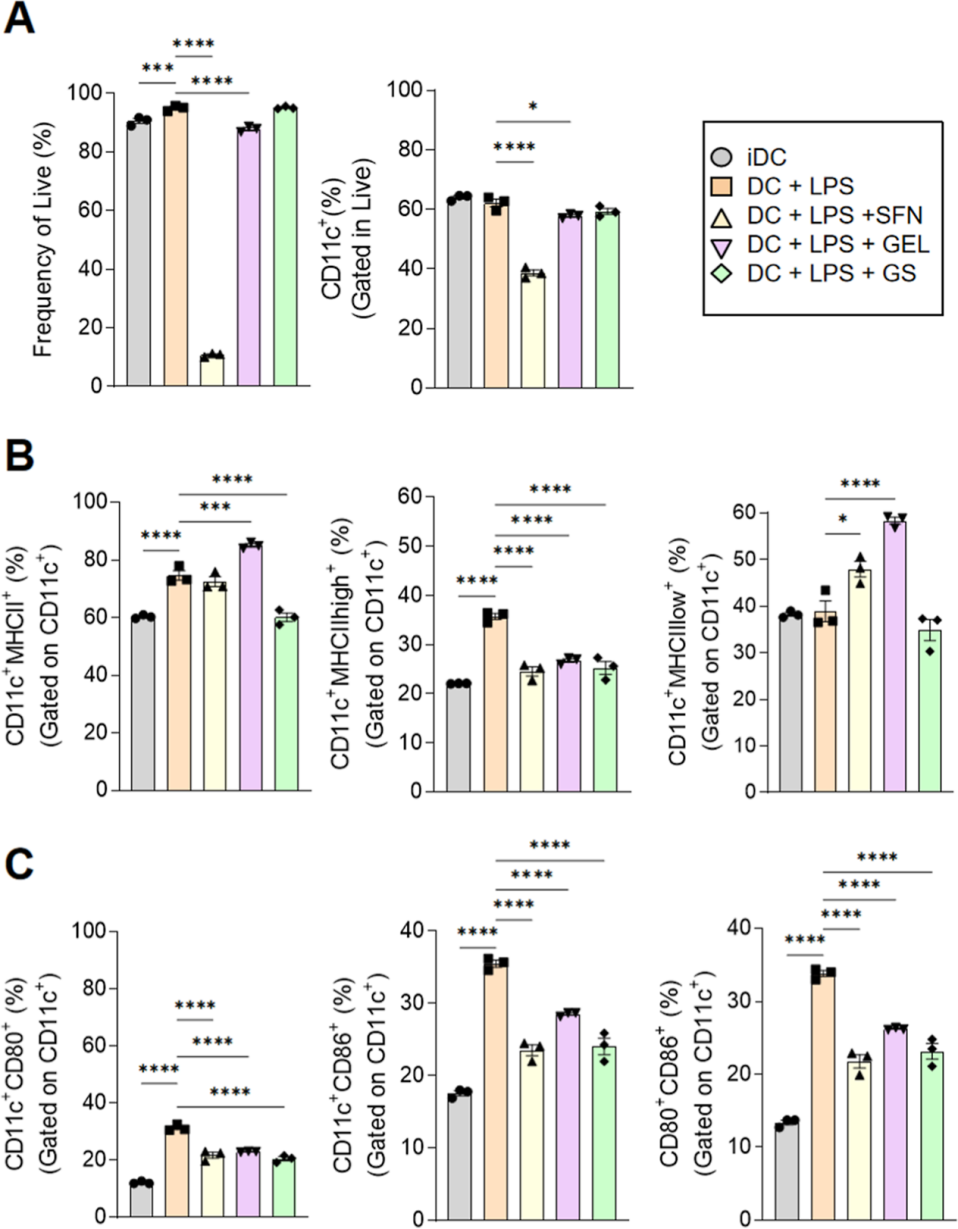
BMDC in vitro treatment with GS change MHCII/CD80/CD86 expression. Bone marrow DC were cultivated for 6 days, treated with SFN, GEL, or GS for 24 h and then left in the presence of LPS (20 ng/mL) for 24 h to be analyzed by flow cytometry. (A) Quantification of live and CD11c^+^ frequency cells. (B) Quantification of CD11c^+^ MHCII^+^ or MHCIIhigh^+^ or MHCIIlow^+^ frequency (gated on CD11c^+^). (C) Quantification of CD11c^+^ CD80^+^ or CD86^+^ or CD80^+^CD86^+^ (gated on CD11c^+^). Data were expressed as mean ± SD. LPS, lipopolysaccharide; iDC, dendritic cell without any treatment; SFN, sulforaphane; GEL, hydrogel (PL407+HA); GS, hydrogel (PL407+HA) with sulforaphane. The Shapiro–Wilk test was performed for normality tests and one-way ANOVA for statistical differences. *p-value ≤0.05; **p ≤ 0.01; ***p ≤ 0.001; ****p ≤ 0.0001.

**Figure 7:**
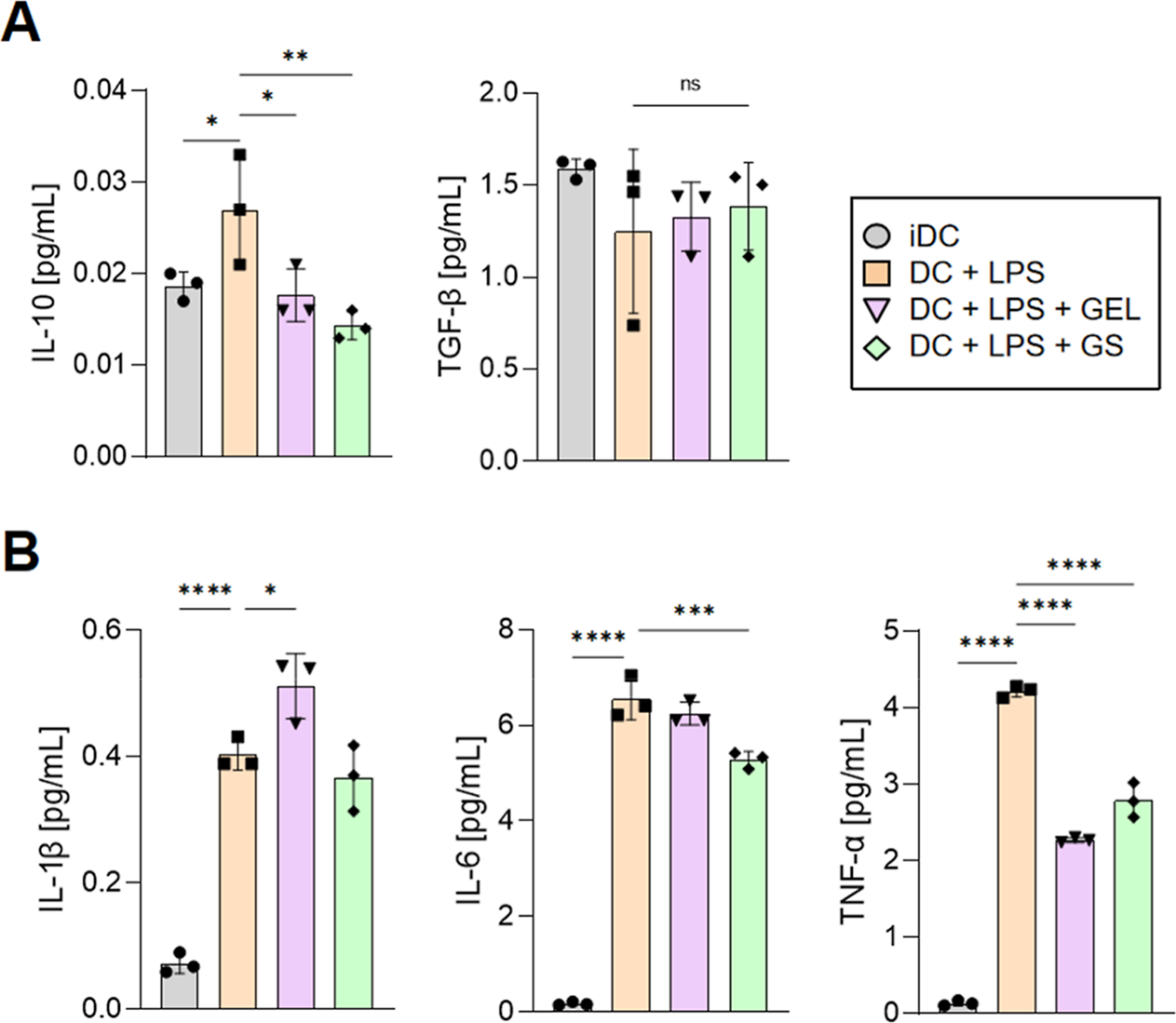
BMDC in vitro treatment with GS reduces IL-10 anti-inflammatory cytokines and reduces IL-6 and TNF-α pro-inflammatory cytokines. Bone marrow DCs were cultivated for 6 days, treated with GEL or GS for 24 h, then left in the presence of LPS (20 ηg/mL) for 24 h, and analyzed by the ELISA kit. (A) Quantification of anti-inflammatory cytokines. (B) Quantification of pro-inflammatory cytokines. Data were expressed as mean ± SD. Representative graphs of 2 independent experiments. The Shapiro–Wilk test was performed for normality tests and one-way ANOVA for statistical differences. NS, non-significant; LPS, lipopolysaccharide; iDC, DC without any treatment; GEL, hydrogel (PL407+HA); GS: hydrogel (PL407+HA) with sulforaphane. *p-value ≤0.05; **p ≤ 0.01; ***p ≤ 0.001; ****p ≤ 0.0001.

From these cell cultures, we analyzed by ELISA the anti- (Figure 7A) and pro-inflammatory (Figure 7B) cytokines to better understand the DC activation in vitro, demonstrating a reduction in the anti-inflammatory cytokine IL-10 and a reduction in pro-inflammatory cytokines IL-6 and TNF-α, while TGF-β and IL-1β showed no difference when compared to the LPS-treated group.

## 3 Discussion

The GS formulation itself has the purpose of controlled drug delivery, in our case, the SFN phytochemical delivery, containing thermosensitive hydrogel (PL407), with HA. This addition changed the values of *G*′ and *G*″; however, the GS formulation presented the greatest difference in the *G*′/*G*″ ratio in relation to isolated PL407, indicating that the hydrogel has a predominant elastic modulus over the viscous modulus, which is important for a s.c. application in vivo and for maintaining the viscoelastic properties of the material,^29,45^ as well as GS 25% presented a sol–gel transition temperature 4 °C lower than GS 20%, showing that the increase in the final polymer concentration reduces Tgel, and GS 25% presented higher shear stress compared to GS 20%, showing that the copolymer concentration interferes in its response to shear, even though both return to the original conformation after the application of stress. Our data corroborate the literature data, since for different poloxamer solutions, when increasing their concentration, the gelation temperature decreases, while the elastic modulus increases.^46–49^ GS 20% also did not show differences in enthalpy variations, showing that even in systems with higher concentrations of PL, the insertion of hydrophilic polymers and small molecules, such as SFN, impacts the micellar organization and the future formation of the hydrogel. These results corroborate previous studies that observed a similar relationship with PL 407 formulations containing HA.^36,50^

The skin transplant went as expected, and since the treatment had not been done before, monitoring was necessary, but it went as expected from other skin transplant models,^51,52^ and the GS treatment prolongs ALO skin graft survival.^53^ The neutrophilic infiltrate present in histological slides suggests a response to acute rejection, since neutrophils and the substances they secrete, such as neutrophil extracellular traps (NET) and neutrophil gelatinase-associated lipocalin, are considered biomarkers of rejection in kidney transplantation^1,54^ and liver transplantation^55,56^ as well as the proportion of neutrophils in relation to lymphocytes is extremely important in predicting acute rejection.^57,58^ Neutrophils are the first line of recognition and defense and have a pro-inflammatory function, facilitating tissue repair,^59^ and in organ transplantation, their role is important both in cellular rejection, increasing alloimmunity and rejection by T lymphocytes, and in humoral rejection, actively participating in tissue damage through their activation by antibodies.^60^ Interestingly, we did not observe differences in IL-17 production in the draining lymph node of animals treated with GS, and IL-17 is a crucial cytokine for neutrophil recruitment.

When we look at DC in dLN, some studies evaluated the effect of tolerogenic DCs (Tol-DCs) in skin transplantation, treated with IL-10 and injected into the recipient, but did not prolong graft survival,^61^ suggesting the need for other stimuli. A meta-analysis revealed that isolated Tol-DCs prolonged graft survival in several transplantation models, with greater efficacy when combined with immunosuppressants.^62^ In a model of skin transplantation from a Balb/c donor to a C57Bl/6 recipient, treated with transfusion of donor spleen cells and the anti-CD154 antibody (anti-CD40 ligand), graft survival was associated with the absence of IFN-γ in the graft, and its long-term maintenance required the continuous presence of CD4^+^ T cells.^63^

These data demonstrate that GS treatment interferes with the activation of DCs in vivo and the differentiation and activation of CD4^+^ T lymphocytes, which may explain the prolongation of graft survival. Considering other inflammatory processes related to the skin, GS was efficient in the treatment of mice in a psoriasis model induced by IMQ, reducing the proportions of Th1 and Th17 cells, and in lupus-like MRL/Lpr mice, increasing life expectancy and decreasing the proportions of plasma cells, follicular T cells, neutrophils, and DCs dLNs.^64^

GS tends to cause an effect on CD4^+^ T lymphocytes and not on CD8^+^ T, probably because the formulation activates the indirect pathway. Due to the nature of antigen processing and presentation, the indirect pathway is directed toward the activation of alloreactive CD4^+^ T lymphocytes, while the direct pathway is more associated with the activation of CD8^+^ alloreactive T lymphocytes.^9^

The serum of the animals treated with GS showed no difference in urea and creatinine concentration when compared with the ALO group, although both were above the maximum value for normal male mice: the urea normal range is 20–40 mg/dL and the serum creatinine level is in the range of 0.3–0.5 mg/dL.^65^ The mean AST value for male mice is 48 U/L (40–60) and ALT is 30 U/L (24–40),^66^ and even in ALO control mice, there was an increase in the levels of these enzymes.

Despite the wide use of poloxamers as drug delivery systems, some controversial aspects of their biosafety are still being discussed in the literature. Especially PL407 has been pointed out as an inducer of hyperlipidemia (by oral or injectable routes, up to 1 g/kg).^67,68^ On the contrary, other authors reported that PL407 daily injections reduced the monocyte migration into atherosclerotic lesions when evaluated in a Ldl −/– mice model.^69^ Other important point to be considered is that Tx can increase liver enzyme levels due to graft injuries such as previous donor transaminase levels,^70,71^ ischemia,^72^ inflammation caused by postischemia reperfusion,^71^ and rejection itself.^73^ Elevated serum aminotransferase enzymes are also common in hematopoietic stem cell transplantation.^74,75^ In this sense, poloxamers can cause dose-dependent hypercholesterolemia and mild nephrotoxicity.^29,68^ However, as reported here, the Tx condition also contributed to those results. These data together demonstrate that our results are consistent with those in the literature.

The response of DC in vitro was observed first, the action of each component of the formulation, so we can say, if there is any death, which component was responsible for it. We observed that the concentrations of SFN alone presented a greater quantity of dead cells when compared to those of other groups (LPS, GEL, GS). SFN is already used in other studies considering adequate and nontoxic concentrations ranging from 1 μM to 40 μM^76–82^ in different types of cells studied and the company from which the SFN came. However, considering our cell type and lineage, studies with high concentration levels^24^ and low concentration levels^83^ used SFN and considered its action efficient, without causing cytotoxicity, suggesting that for our cultures, the micellar structure contains the toxicity of isolated SFN and presents good viability.

While the promising and consistent results across replicates suggest that the observed effects are robust, this study is subject to some limitations that warrant consideration. First, the relatively small sample size in some groups may limit the generalizability of our conclusion. Second, the impact of the HA component itself on the AR was not addressed. Addressing this will be crucial to fully uncover the precise effects of both SFN and HA on AR. Third, although observing the SFN-loaded hydrogel’s impact on in vitro BMDC cell culture provides a controlled system for evaluating the early mechanisms of DC activation, this approach does not fully replicate the complex in vivo immune response. Finally, we did not evaluate the long-term consequences for SFN treatment or its potential impact on other transplant outcomes, such as retransplantation or application in other organ types (e.g., liver, kidney, or nonsolid organ transplantation). Future studies incorporating a larger cohort and evaluating a broader range of transplantation outcomes are necessary to fully determine the potential clinical benefit of SFN treatment and to validate the current findings.

## 4 Conclusion

The hydrogel formulation, composed of SFN (0.1%) and HA (0.5%) dispersed in a PL 407 matrix (20% w/v), presented a liquid-viscous structure suitable for its s.c. in vivo and in vitro application. In vitro treatment with GS in BMDC cultures did not demonstrate cytotoxicity to cell viability. After in vivo treatment on the 9th day post Tx, 100% of untreated mice rejected the Tx, and treatment with s.c. injections of GS every 3 days increased allograft survival (80%) for 14 days (*p* < 0.001), demonstrating the efficiency of treatment with GS. Furthermore, after 5 days of Tx, in the dLN analysis, the treated group presented modulation of the immune system, with a reduction in the frequency of DC, naïve CD4^+^, and effector memory and central memory T cells, and there was a tendency for a decrease in IFNγ-producing CD4^+^ T cells. The need for greater guidance on the deeper mechanisms involved in the immunological response to GS treatment suggests further research into direct therapies. We can conclude that GS treatment alleviates the activation of immune cells in vivo, preventing AR and contributing to increasing allograft survival.

## 5 Experimental Section

### 5.1 Hydrogel Preparation

For hydrogel synthesis, 20% or 25% (w/v) PL 407 (Pluronic F-127, Sigma-Aldrich, Chem. Corp. Saint Louis, MO, USA) solutions were prepared in ultrapure water and kept under constant magnetic stirring (300 rpm) in an ice bath until complete dissolution, and a transparent solution was formed. HA (150 kDa; Viafarma Ind. Farmc., São Paulo, Brazil) in powder (0.5%) and SFN (0.1% SFN, Toronto Research Chemicals, Ontario, CA) were incorporated into the PL solutions under the same conditions as previously described. All samples were stored at 8 °C until convenient use.

### 5.2 Hydrogel Physico-Chemical Characterization

#### 5.2.1 Differential Scanning Calorimetry and Rheological Assays

DSC assays were performed on a DSC 214 Polyma calorimeter (NETZSCH, DSC21400A-0187-L, Germany). The hydrogel samples were weighed directly in an aluminum pan and analyzed according to three successive thermal cycles of heating and cooling (0–50 °C), at a rate of 5 °C/min, using an empty pan as a reference. All measurements were executed in triplicate, and the results are represented in Table S1, by heat flux (J/g) versus temperature (°C).

The rheological analysis was carried out in an oscillatory cone–plate geometry rheometer (Kinexus Lab., Malvern Instruments, Malvern, UK) for determining mechanical parameters such as the elastic modulus (*G*′), viscous modulus (*G*″), viscosity (η), and sol–gel transition (Tsol–gel). Hydrogel samples (1 mL) were analyzed under a temperature interval from 10 to 50 °C and a frequency of 1 Hz. Additionally, frequency sweep analysis (0.1 to 10 Hz) was also performed at 37 °C and a shear stress of 2 Pa. Data were analyzed by rSpace for Kinexus software.

#### 5.2.2 In Vitro Assays

In vitro SFN release assays were carried out by a vertical diffusion cell system, Franz-type, with an area of 1.72 cm^2^ (Microette Plus; Hanson Research, USA). The donor and recipient compartments were separated by cellulose acetate membranes of MWCO (1000 Da). The formulations were placed in the donor compartment, 1 mL, while from the recipient compartment (containing 5 mM sodium phosphate buffer in 154 mM NaCl, pH 7.4, 37 °C, under shaking at 350 rpm), 1 mL aliquots were collected at intervals of 30 min, 1 h, 2 h, 4 h, 8 h, 12 h, 24 h, 36 h, 48 h, and 72 h and analyzed by HPLC for SFN quantification.

In vitro SFN dissolution assays were performed in a system consisting of donor and receptor compartments. The donor compartment (cylinder 0.8 × 2.0 × 2.5 cm) was filled with 1 mL of the formulation, while the recipient compartment was filled with buffer solution (5 mM sodium phosphate buffer in 154 mM NaCl, pH 7.4), characterizing a dissolution model without the use of a limiting membrane. The entire system was maintained under magnetic stirring at 350 rpm, 37 °C. Aliquots of 1 mL were removed at intervals of 30 min, 1 h, 2 h, 4 h, 8 h, 12 h, 24 h, 36 h, 48 h, and 72 h and analyzed by HPLC for drug quantification (the equation of the straight line was *y* = 0.03242 + 0.5116*x*, the coefficient of determination was 0.9891, the detection limit was 0.0497 μg/mL, and the quantification limit was 0.134 μg/mL).

Both profiles were analyzed considering the zero-order, Higuchi, Korsmeyer–Peppas, and Hixson–Crowell models, as described by equations reported in Supporting Information #1.^84^

### 5.3 Animals and Cells

#### 5.3.1 Animals

All experiments were conducted in adherence with the Brazilian Committee for Experimental Animals and were approved by the Institutional Ethics Committee on Animal Use of the Federal University of ABC, Santo André-SP, Brazil, by number 3694011119. Male Balb/c mice (MHC H-2^b^, 6 to 8 weeks old) were skin donors, and male C57BL/6 mice (MHC H2^d^, 6 to 8 weeks old) were Tx recipients, both purchased from Bioterio of Federal University of ABC.

A total of 5 male Balb/c mice and 30 (4–5 mice/group) male C57BL/6 mice were used in the work. The animals were maintained under specific pathogen-free conditions in groups of five and, after transplant, one in a box, in a room with a 12 h/12 h light/dark cycle at 22 °C at the Federal University of ABC, receiving water and food ad libitum.

#### 5.3.2 In Vivo Skin Transplant Model

A fully mismatched skin Tx model was performed by implanting a skin donor from Balb/c animals into C57BL6 recipients. Briefly, Balb/c mice were euthanized by intraperitoneal (ip) anesthetic overdose, and their backs were completely shaved. The tissue on the back was removed and collected, with the aid of scissors, and the skin was cut using a scalpel into similar sizes (approximately 1 cm^2^) and kept in cold PBS until Tx. In parallel, C57BL/6 recipient mice were anesthetized via ip, their backs were shaved, and the area was aseptically prepared with 70% ethanol. Next, an area equivalent to the size of the donor’s skin, forming a “place”, was removed from the recipient and replaced with the donor’s graft, followed by suturing with a 4–0 silk thread using a triangular needle (Shalon® Suturas, Brazil). The mice were divided into the following groups: ISO, isogenic transplant, control of the transplant, there is no rejection; ALO, allogeneic transplant, there is rejection, untreated group; ALO-GS, allogeneic transplant, there is rejection, treated group. Treatment with GS in vivo occurred by subcutaneous injection (s.c.), at a dose of 200 μL/formulation once every 3 days post Tx. The Tx and graft survival were monitored and evaluated daily after surgery by visual (photos) and tactile monitoring of the Tx until the day of euthanasia. Graft loss was considered when the proportion of necrosis was equal to 100%.^53,85^ After euthanasia, the grafts, the spleen, and the axillary and brachial lymph nodes—draining lymph nodes (dLN)—were collected and processed for flow cytometry analysis, while the skin Tx was evaluated by histological analysis.

#### 5.3.3 Histological and Serum Evaluation

The skin graft samples were fixed in buffered paraformaldehyde (4%) for 24 h, dehydrated, and diaphanized for paraffin. The paraffin blocks were cut into 5 μm sections, and the sections were deparaffinized in xylene, hydrated, and stained with hematoxylin and eosin (HE). Microscopic analysis of the graft was performed blindly by a pathologist. The images were analyzed, and the intensity of the reaction was classified qualitatively and semiquantitatively, based on the percentage of inflammation infiltrated caused by the cell types present in the image slide, considering the classification as mild (when up to 25% of the analyzed variable was present in the slide), moderate (50% of the analyzed variable present), and severe (more than 75% of the slide containing the analyzed variable).

Serum was used to assess renal function (urea and creatinine) as well as liver function (aspartate aminotransferase (AST) and alanine aminotransferase (ALT)).

Serum analysis was performed for AST and ALT according to the manufacturer’s instructions (Labtest, Brazil, category #52 and category #53, respectively), using the Reitman and Frankel method. Absorbances were determined at 505 nm, zeroed with distilled or deionized water. A reference curve according to the kit was used.

For urea and creatinine measurement, samples were deproteinized as follows: 100 μL of serum was added to a mix containing 100 μL of 1.84% sulfuric acid, 100 μL of 10% sodium tungstate, and 200 μL of water. The samples were centrifuged at 10,000 rpm for 10 min, and the supernatant was collected. Serum creatinine was measured using Jaffé’s method, and serum urea was measured using the urea kit (Labtest, Brazil) according to the manufacturer’s instructions. Absorbance were determined at 520 nm together with a standard curve on a Synergy HT spectrophotometer (Biotek).

#### 5.3.4 Flow Cytometry

Flow cytometry data were acquired on the FACSCanto II cytometer (BD Biosciences, USA) and analyzed with FlowJo software (BD Biosciences, USA). For detection of intracellular cytokines, 1 × 10^6^ cells were stimulated with 50 ng/mL phorbol 12-myristate 13-acetate (PMA) and 1 μg/mL ionomycin in the presence of 1 μg/mL brefeldin (BioLegend) for 4 h in complete IMDM medium (37 °C with 5% CO2). Cells were surface-stained (25 μL/well antibody) and underwent fixation and permeabilization with the Cell Permeabilization Kit reagent (Life Technologies) following the manufacturer’s protocol, followed by staining for intracellular cytokines. For surface marker detection, 1 × 10^6^ cells were incubated in 25 μL of a mix of PBS +2% fetal bovine serum (FBS) (FACS Buffer) containing the antibodies for 30 min at 4 °C in the dark. The following antibody clones (BioLegend) were used for staining cells: CD4 (RM4-5), CD8α (53–6.7), CD11b (M1/70), CD11c (N418), CD44 (IM7), CD62L (MEL-14), CD80 (16-10A1), CD86 (GL-1), IA-IE (M5/114.15.2), IFNγ (XMG1.2), IL-17A (TC11-18H10.1), TCRβ (H57-597), and Zombie NIR were used to identify live/dead cells for all experiments.

#### 5.3.5 DC Culture and ELISA

BMDC were obtained by differentiating bone marrow cells collected from mice (C57BL/6).^86^ Briefly, femurs and tibias were collected and placed with the distal end facing downward; the epiphyses were cut before positioning them into 10 μL tips inserted into 1.5 mL microtubes, following centrifugation. Afterward, the tips containing the bones were discarded, and the pellet was suspended in PBS, filtered through a 70 μm cell strainer, and treated with lysis buffer (155 mM NH_4_Cl, 10 mM NaHCO_3_, and 0.1 mM EDTA, pH 7.2). Dead cells were excluded by the trypan blue assay, and 2 × 10^6^ viable cells were cultured in flat-bottom 6-well plates in 2 mL of IMDM medium with 10% fetal bovine serum (FBS), 1% penicillin/streptomycin, and 1% pyruvate, in the presence of 20 ng/mL Granulocyte and Macrophage Colony Stimulating Factor (GM-CSF)^87^. On day 3 of cultivation, 50% of the medium volume was exchanged, and a concentration of 50% of the volume was added to complete IMDM medium with the concentration of GM-CSF for the entire volume. The cells in the culture were maintained at 37 °C and 5% CO_2_. On day 6, the cells were treated with SFN, GEL, or GS for 24 h and then left in the presence of LPS (20 ng/mL) for 24 h to be analyzed by flow cytometry.

The supernatant from the DC culture was tested for IL-10, IL-1β; TNF-α; IL-6, TGF-β concentration by ELISA (R&D Systems), following the manufacturer’s instructions.

Cell cytotoxicity assays were performed with the GS formulation or isolated SFN, exposed in direct contact with DC lines, for 24 h; after this period, the cells were labeled with antibodies and evaluated by flow cytometry.

#### 5.3.6 Statistical Analysis

The results are presented as the mean ± standard deviation. Experimental data were analyzed by GraphPad Prism Software 9 (GraphPad Software Inc., La Jolla, CA, USA). Normality tests were performed using the Shapiro–Wilk test, and statistical differences were assessed using the unpaired *t*-test and one-way ANOVA (paired tests), followed by Tukey’s post hoc test or Kruskal–Wallis (unpaired tests), followed by Dunn’s post hoc test. Statistical significance was defined as *p* ≤ 0.05. Considering different significance levels, asterisks were used as follows: *p-value ≤0.05; **p-value ≤0.01; ***p-value ≤0.001; ****p-value ≤0.0001.

## Supplementary Material



## Notes

● Physicochemical parameters; mathematical models used for analysis; release constant and correlation coefficient; cell viability in vitro; frequency of T CD8^+^ lymphocyte population; frequency of IFN-γ- or IL-17-producing CD8^+^ T cells; urea and creatinine levels; and AST/ALT levels (PDF)

## Abbreviations

APC: antigen presenting cell
AR: acute rejection
BMDC: bone marrow-derived dendritic cells
DC: dendritic cells
dLN: draining lymph node
GEL: hydrogel (PL407 + HA)
GS: hydrogel (PL407 + HA) with sulforaphane
HA: hyaluronic acid
ip: intraperitoneal injection
LPS: lipopolysaccharide
MHC: major histocompatibility complex
PL: poloxamers
s.c.: subcutaneous injection
SFN: sulforaphane
Tx: transplantation
