# Supplementary material for: Sulforaphane-Loaded Hydrogel Prolongs Fully MHC-Mismatched Skin Allograft Survival
Source: ACS Appl Bio Mater. 2025 Oct 29;8(11):9780–93. doi: 10.1021/acsabm.5c01022 (PMC12628332; doi:10.1021/acsabm.5c01022)
Supplement: Supplementary file 1 [file mt5c01022_si_001.pdf]

Supporting Information

Sulforaphane-loaded hydrogel prolongs fully MHC-mismatched skin allograft survival

Lorena Doretto-Silva[a], Laura Quadros-Pereira[a], Anderson F. Sepulveda[b], Victor Y. Yariwake[a,c], José A. O. Nery-Neto[a,c], Eloisa M. da Silva[a,d], Isabela L. Doretto[e], Niels O. S. Câmara[c], Daniele R. de Araujo*[b,f], Vinicius Andrade-Oliveira*[a]

[a] Mucosal Health and Immunity Laboratory (MHIL), Center for Natural and Human Science, Federal University of ABC, Santo André, São Paulo, Brazil;
[b] SISLIBIO, Center for Natural and Human Science, Federal University of ABC, Santo André, São Paulo, Brazil;
[c] Institute of Biomedical Sciences, University of São Paulo, SP, Brazil
[d] Paulista School of Medicine, Federal University of São Paulo, São Paulo, SP, Brazil;
[e] Agronomy and Veterinary Medicine School, University of Brasília, Brasília, Brazil
[f] Department of Biophysics, Paulista Medical School, Federal University of São Paulo, São Paulo, SP, Brazil.

Corresponding authors
*Email: andrade.vinicius@ufabc.edu.br (V. Andrade-Oliveira)
*Email: daniele.ribeiro@unifesp.br (D. R. de Araujo)

Table S1. Physicochemical parameters relative to formulations in the presence or absence of HA and SFN, with temperature of micellization (Tm), and enthalpy variations (ΔH) related to the micellization process. Also, it is pointed and elastic (G′), viscous (G″) moduli, sol-gel transition temperatures (Tsol-gel), and viscosity (η. mPa·s) at 25 °C and 37 °C

Formulation (% w/v)	Additives (% w/v)	Tm (ºC)	ΔH (J/g)	G' (Pa)	G" (Pa)	G'/G" (1HZ)	T sol-gel	η (mPa s) 25ºC (x10^{2})	η (mPa s) 37ºC (x10^{6})
PL407 20	-	20.4	-2.467	3913	697.5	23.79	27.48± 0.016	1.5	1.9
	HA 0.5	20.4	-3.045	1339	263.9	17.38	27.34± 0.014	4.1	1.4
	SFN 0.1	21.1	-2.135	3353	807.9	21.69	27.12± 0.014	1.5	2.0
	HA 0.5 SFN 0.1	20	-2.767	3000	544.4	14.65	27.33± 0.016	4.8	1.5
PL407 25	-	18.8	-3.101	5555	1321	31.89	23.19± 0.014	22700	3.2
	HA 0.5	18.3	-3.653	6908	1598	37.94	23.24± 0.016	18140	2.5
	SFN 0.1	18.6	-2.576	9161	1082	30.68	23± 0.014	23290	3.2
	HA 0.5 SFN 0.1	18.2	-2.515	6906	1067	25.12	23.06± 0.018	16870	2.4

Supporting Information #1

For the evaluation of *in vitro* release and dissolution profiles, it is necessary to consider the mathematical modeling of the distribution of these profiles (Siepmann, J., & Siepmann, F. 2008), since we have different methods, one has a separating membrane and the other has free access. Considering the properties of the formulation, the mathematical models used for analysis were (Costa, P., & Sousa Lobo, J. M. 2001):

- Zero-order kinetics - the dissolution of the drug from pharmaceutical forms that do not disintegrate and release the drug slowly:

Equation $Q_t = Q_0 + K_0^t$
where Qt is the amount of drug dissolved in time t, Q0 is the initial amount of drug in the solution and K0 is the zero-order release constant.

- Higuchi - the release rate is linear as a function of the square root of time and the drug is the only component that diffuses through the medium:

Equation $Q_t = K_H^{t1/2}$
where Q is the concentration of drug released/permeated as a function of time, K is the release constant (diffusion coefficient) and t is time.

- Hixson-Crowell - recognizes that the regular area of the particle is proportional to the cubic root of its volume:

Equation $Q_0^{1/3} - Q_t^{1/3} = K_s.^t$
where, Q0 is the initial amount of drug in the pharmaceutical form; Qt is the remaining amount of drug in the pharmaceutical form at the end of time t; and KS is the Hixson-Crowell release constant.

- Korsmeyer-Peppas - correlates drug release as a function of time by an exponential equation:

Equation $M_t/M_\infty = kt^n$
where Mt is the amount of drug released in a given time t, M∞ is the amount of drug released in an infinite time, k is the release kinetic constant and n is the release exponent

Table S2. Release constant (K) values with error, correlation coefficient for SFN release from release and dissolution tests

Formulation			GS 20%	GS 25%
**Dissolution**	**Zero-Order**	K	2.25	2.26
		K error	0.21	0.21
		R^2	0.93	0.93
	**Higuchi**	K	20.96	21.02
		K error	0.68	0.82
		R^2	0.99	0.99
	**Hixson-Crowell**	K	0.05	0.04
		K error	0.01	0.01
		R^2	0.71	0.72
	**Korsmeyer Peppas**	K	0.62	0.44
		K error	0.06	0.02
		R^2	0.94	0.98
		n	4.15	4.44
**Release**	**Ordem zero**	K	3.64	3.13
		K error	0.91	1.02
		R^2	0.70	0.57
	**Higuchi**	K	35.02	30.25
		K error	9.03	8.27
		R^2	0.75	0.69
	**Hixson-Crowell**	K	0.04	0.03
		K error	0.02	0.01
		R^2	0.57	0.53
	**Korsmeyer Peppas**	K	0.82	0.51
		K error	0.19	0.10
		R^2	0.78	0.82
		n	4.00	4.57

Figure S1

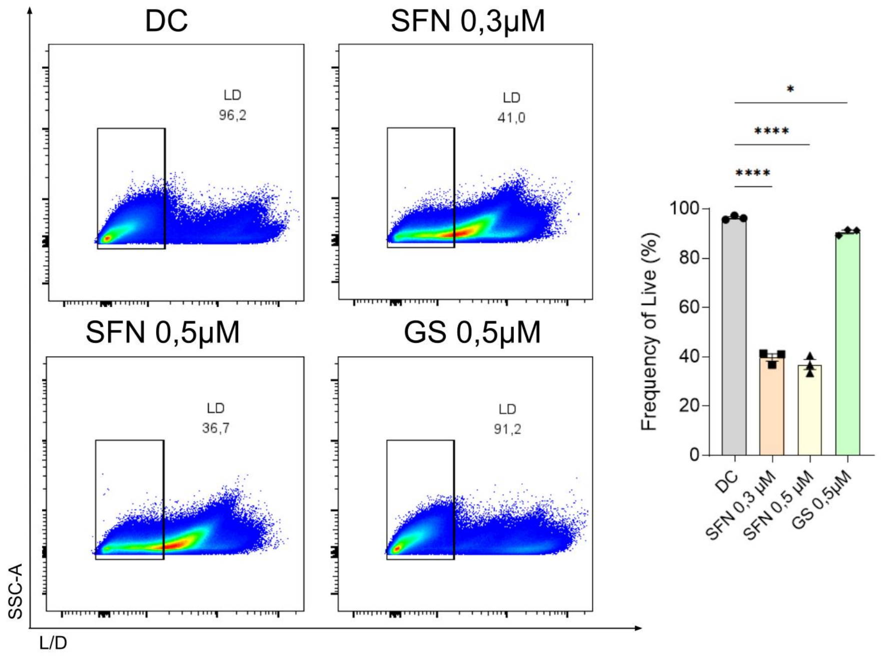

Figure S1. Cell viability of GS and SFN in vitro. (Left) Flow citometry intensity graphs representing the percentage of live cells during treatment at different doses of SFN alone (0,3 and 0,5 μM) or with GEL (SFN 0,5 μM) in BMDC culture. (Right) Frequency of BMDC viability from cytometry analysis. BMDC: bone marrow dendritic cell; DC dendritic cel without any treatment; SFN: sulforaphane; GS: hydrogel (PL407+HA) with SFN. Data were expressed as mean ± SD of triplicate. * p-value ≤ 0.05; **** p < 0.0001.

Figure S2

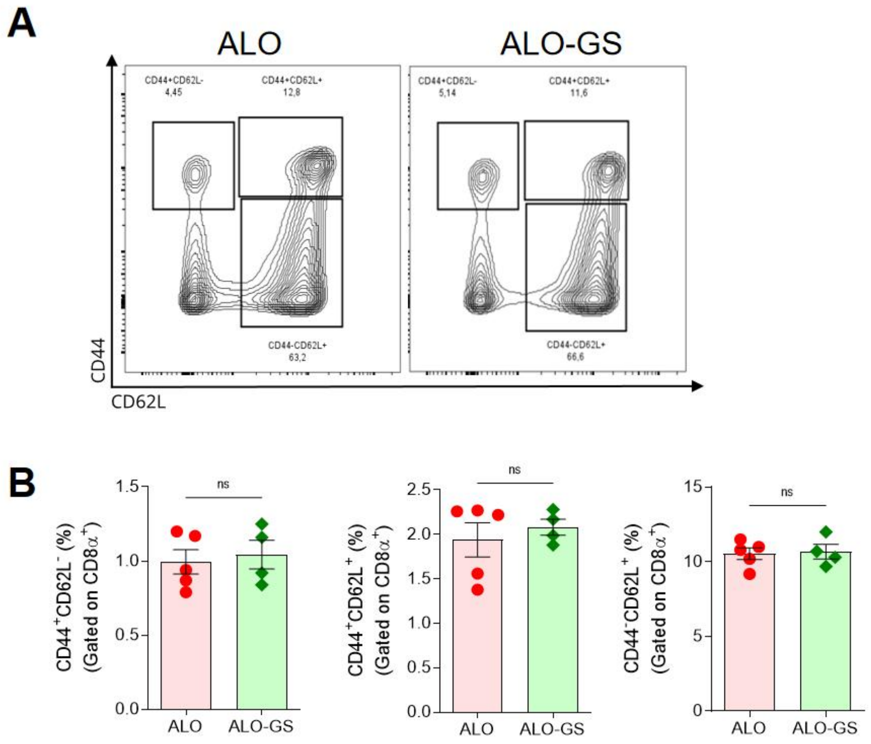

Figure S2. GS treatment does not reduce the frequency of T CD8 lymphocyte population in the dLN 5 days post-Tx. (A) Representative cytometry panel for frequency of parent and (B) frequency of effector memory ($CD44+CD62L^-$), central memory ($CD44^+CD62L^+$) and naïve T cells ($CD44^-CD62L^+$) from $CD8^+$ T lymphocytes. Shapiro-Wilk test was performed for normality tests and unpaired T-test for statistical differences. dLN - draining lymph node.

Figure S3

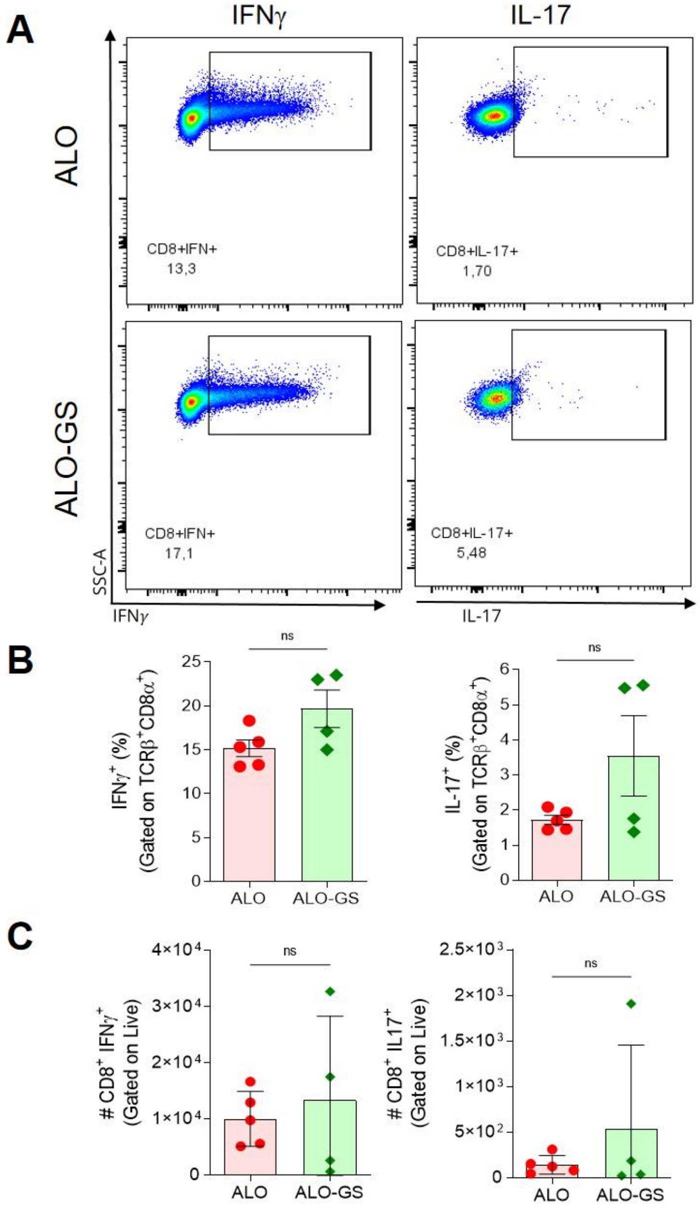

Figure S3. GS treatment does not reduce the frequency of IFN-γ- or IL-17-producing $CD8^+$ T cells in the dLN 5 days post-Tx. (A) Representative cytometry panel of the frequency of $IFN\gamma^+$ and $IL\text{-}17^+$ $CD8^+$ T (gated on $TCR\gamma^+CD8^+$ cells). (B) Quantification of the frequency of production of cytokines IFNγ and IL-17 according to the values in (A). (C) Absolute number of $CD8^+$, $CD8^+IFN\gamma^+$ or $CD8^+IL\text{-}17^+$ T cells relative to (B) Shapiro-Wilk test was performed for normality tests and unpaired T-test for statistical differences. NS – not significant; IFNγ -interferon gamma; IL-17 - interleukin 17; SSC-A - side scatter; dLN - draining lymph node.

Figure S4

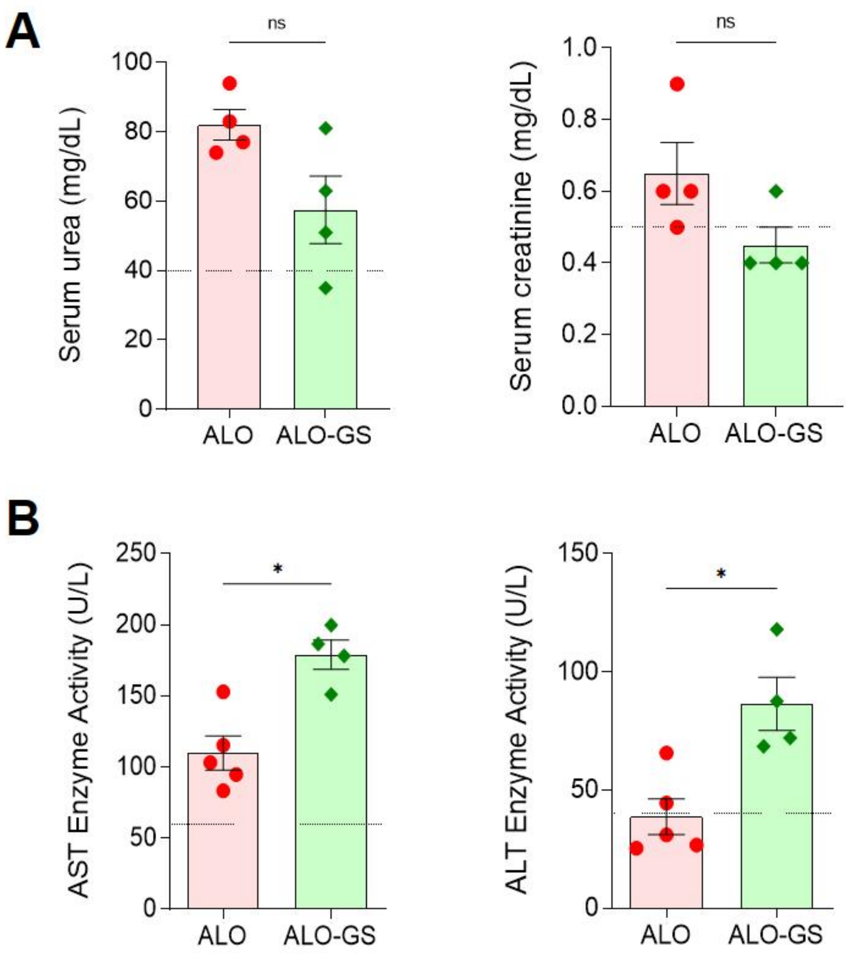

Figure S4. GS treatment does not show cytotoxicity in other organs. In day 5 pos-Tx, during euthanasia, the serum was collected and analised for (A) Urea and creatinine levels. (B) AST/ALT levels. Dotted line represents the maximum reference value for that analyte. Shapiro-Wilk test was performed for normality tests and unpaired T-test for statistical differences. AST - aspartate aminotransferase; ALT - alanine aminotransferase.
